# Glucocorticoid receptor gene polymorphisms do not affect growth in fetal and early postnatal life. The Generation R Study

**DOI:** 10.1186/1471-2350-11-39

**Published:** 2010-03-03

**Authors:** Miranda JJ Geelhoed, Eric AP Steegers, Jan W Koper, Elisabeth FC van Rossum, Henriette A Moll, Hein Raat, Henning Tiemeier, Albert Hofman, Vincent WV Jaddoe

**Affiliations:** 1 The Generation R Study Group, Erasmus Medical Center, Rotterdam, The Netherlands; 2 Department of Epidemiology, Erasmus Medical Center, Rotterdam, The Netherlands; 3 Department of Pediatrics, Erasmus Medical Center, Rotterdam, The Netherlands; 4 Department of Obstetrics & Gynecology, Erasmus Medical Center, Rotterdam, The Netherlands; 5 Department of Internal Medicine, Erasmus Medical Center, Rotterdam, The Netherlands; 6 Department of Public Health, Erasmus Medical Center, Rotterdam, The Netherlands; 7 Department of Child and Adolescent Psychiatry, Erasmus Medical Center, Rotterdam, The Netherlands

## Abstract

**Background:**

Glucocorticoids have an important role in early growth and development. Glucocorticoid receptor gene polymorphisms have been identified that contribute to the variability in glucocorticoid sensitivity. We examined whether these glucocorticoid receptor gene polymorphisms are associated with growth in fetal and early postnatal life.

**Methods:**

This study was embedded in a population-based prospective cohort study from fetal life onwards. The studied glucocorticoid receptor gene polymorphisms included *Bcl*I (rs41423247), *TthIII*I (rs10052957), GR-9β (rs6198), N363S (rs6195) and R23K (rs6789 and6190). Fetal growth was assessed by ultrasounds in second and third trimester of pregnancy. Anthropometric measurements in early childhood were performed at birth and at the ages of 6, 14 and 24 months postnatally. Analyses focused on weight, length and head circumference. Analyses were based on 2,414 healthy, Caucasian children.

**Results:**

Glucocorticoid receptor gene polymorphisms were not associated with fetal weight, birth weight and early postnatal weight. Also, no associations were found with length and head circumference. Neither were these polymorphisms associated with the risks of low birth weight or growth acceleration from birth to 24 months of age.

**Conclusions:**

We found in a large population-based cohort no evidence for an effect of known glucocorticoid receptor gene polymorphisms on fetal and early postnatal growth characteristics. Further systematic searches for common genetic variants by means of genome-wide association studies will enable us to obtain a more complete understanding of what genes and polymorphisms are involved in growth in fetal life and infancy.

## Background

Glucocorticoids are important regulators of growth, development and metabolism. The effects of these hormones, including cortisol, are mediated by glucocorticoid receptors. The sensitivity to glucocorticoids is known to show a large interindividual variation [1]. Polymorphisms in the glucocorticoid receptor gene have been suggested to contribute to this difference in sensitivity and thereby to differences in growth, development and metabolism. These glucocorticoid receptor gene variants may also explain part of the previously demonstrated associations between growth characteristics in early life and metabolic disease, including type 2 diabetes, in adult life [2,3].

Four different variants in the glucocorticoid receptor gene have been described to be associated with cortisol sensitivity [4-6]. Several studies analyzed the associations of these glucocorticoid receptor gene variants with body composition and obesity in adults. The R23K variant (two Single Nucleotide Polymorphisms in complete linkage disequilibrium) was found to be associated with higher serum cortisol concentrations as well as a smaller decrease in cortisol after dexamethasone suppression tests. Furthermore, carriers showed lower fasting insulin levels and lower LDL cholesterol levels. These data suggest that carriers of the 23K variant of the R23K polymorphism are relatively more cortisol resistant than non-carriers, which results in a better metabolic health profile in adults [7,8]. By contrast, the *Bcl*1 and N363S polymorphisms were found to cause the opposite effects [9-11]. In healthy subjects over 55 years, these polymorphisms were associated with hypersensitivity to glucocorticoids, resulting in an increased body mass index (BMI) [10]. In addition, a large meta-analysis performed in almost 6,000 individuals concluded that there is no compelling evidence that the N363 polymorphism of the GR-gene is associated with either average BMI or obesity risk [12]. However, results were not consistent [13,14]. The *TthIII*I polymorphism was associated with elevated diurnal cortisol levels, but not with any anthropometric or glucose related phenotype [15]. Recently, the GR-9β polymorphism was found to be related to a decreased sensitivity to glucocorticoids, leading to an increased risk of cardiovascular disease [16]. These results suggest that common functional variants of the glucocorticoid receptor gene may affect body composition. However, apart from the R23K variant, the exact mechanisms have not been confirmed. Also, thus far no studies did assess the effects of these glucocorticoid receptor gene polymorphisms in young children. However, although some studies showed an increasing effect of genetic variants in the glucocorticoid receptor gene with advancing age [8,17], the effect of these glucocorticoid receptor gene polymorphisms might be stronger on anthropometric measures in early life than on body mass index in adult life, because of very limited life style influences.

We hypothesised that genetic variants leading to increased glucocorticoid sensitivity are associated with fetal growth retardation and postnatal growth acceleration. This would be in line with well-known associations of high cortisol exposures with low birth weight and higher postnatal weight. Therefore, we studied in a population-based prospective cohort study from fetal life until the age of 2 years the effects of the *Bcl*I, *TthIII*I, GR-9β, N363S and R23K polymorphisms on anthropometrics in second and third trimester of pregnancy, at birth and postnatally until the age of 24 months.

## Methods

### Design

This study was embedded in the Generation R Study, a prospective cohort study from early fetal life onwards. This study is designed to identify early environmental and genetic determinants of growth, development and health from fetal life until young adulthood and has been described previously in detail [18,19]. Fetal and postnatal growth and their main determinants were repeatedly measured by physical examinations, fetal ultrasounds and questionnaires. We have previously shown that of all eligible children born in the study area 61% participated in the study [19]. The study has been approved by the Medical Ethics Committee of the Erasmus Medical Center, Rotterdam. Written informed consent was obtained from all parents.

### Population for analysis

Analysis were restricted to Caucasian children (n = 4,527) and of whom DNA was available for genotyping (n = 2,839). Reasons for non-availability of DNA were mainly due to logistical constraints at birth, including a relatively large number of home deliveries in the Netherlands and time constraints during hospital deliveries. Fetal growth measurements were available in 2,746 and 2,791 children in second and third trimester of pregnancy, respectively. A total of 81% (n = 2,274), 75% (n = 2,136) and 68% (n = 1,929) participated in the postnatal assessments at the ages of 6, 14 and 24 months. Information about anthropometrics of at least one of the postnatal visits was available in 2,414 subjects of whom 72%, 91% and 100% had measurements at least three, two and one visit. In total, analyses were based on more than 6,000 measurements.

### Genotyping

**Figure 1:**
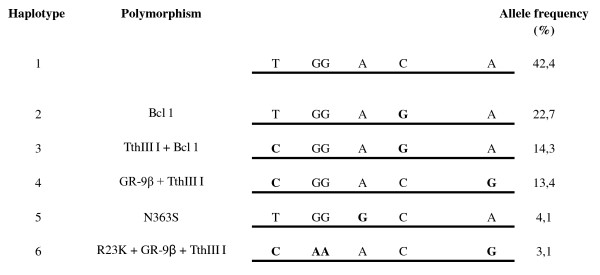
**Schematic overview of the glucocorticoid receptor gene polymorphisms and haplotypes**. Haplotypes are numbered in order of decreasing frequency. The nucleic acid changes are indicated; C = Cytidine, G = Guanine, A = Adenosine, T = Thymine.

DNA was collected from cord blood samples at birth. All participants were genotyped for five known glucocorticoid receptor gene polymorphisms which are known to be associated with changes in glucocorticoid sensitivity or diurnal cortisol levels: *Bcl*I (rs41423247), *TthIII*I (rs10052957), GR-9β (rs6198), N363S (rs6195) and R23K (rs6189 and 6190) [4,5]. The five SNPs used in this study were chosen on the basis of their reported functionality, and not as tagging SNPs, and the purpose was not to determine the level of variation in the GR gene. However, we used four of these five SNPs as tagging SNPs for the GR gene in the program HaploView. The program showed that these four SNPs identify one third (19 out of 57) haplotypes. Only four SNPs could be analysed this way because the fifth (rs41423257), coincidentally the SNP with the highest minor allele frequency, is not in the HapMap database. This result suggests that a considerable part of the genetic variation in the GR gene is captured by these five SNPs. The SNPs used here are all within one LD-block [20]. This block also includes the (proximal) promoters and exon 1 variants 1D, 1E, 1B, 1F, 1C and 1H. It also includes the GR CpG island. The only exon 1 variant not included is 1A. The majority of transcripts contain either 1C or 1B. Figure 1 schematically shows the specific nucleotide variations and allele frequencies of these polymorphisms. Genotyping of the five glucocorticoid receptor gene polymorphisms was performed using Taqman allelic discrimination assay (Applied Biosystems, Foster City, CA) and Abgene QPCR ROX mix (Abgene, Hamburg Germany). The genotyping reaction was amplified using the GeneAmp^® ^PCR system 9600 (95°C (15 minutes), then 40 cycles of 94°C (15 seconds) and 60°C (1 minute)). The fluorescence was detected on the 7900HT Fast Real-Time PCR System (Applied Biosystems) and individual genotypes were determined using SDS software (version 2.3, Applied Biosystems). Genotyping was successful in 97-99% of the samples for the five genotypes. To confirm the accuracy of the genotyping results 276 randomly selected samples were genotyped for a second time with the same method. The error rate was less than 1% for all genotypes. We used the genotype data for each of the 5 polymorphisms to infer the haplotypes present in the population using the program PHASE, which implements a Bayesian statistical method for reconstructing haplotypes from population genotype data [21]. Instead of individual polymorphisms, we studied the haplotype structure of the glucocorticoid receptor gene to encompass a major proportion of variation in the gene. We excluded very rare polymorphisms because they have potential to explain only a very small fraction of variation in response to glucocorticoids seen between individuals. For each haplotype, 3 genotype combinations were distinguished as carrying 0, 1, or 2 copies of the haplotype allele. Haplotype 1 carries the major alleles of the polymorphisms; therefore, the reference allele is defined as carrying 2 copies of haplotype 1. Genotype and allele frequencies were in Hardy Weinberg equilibrium (p > 0.01).

### Growth measurements

#### Fetal growth characteristics

Fetal ultrasound examinations were carried out during visits at the research centers. The respective median (95% range) gestational ages for these visits were 12.6 weeks (9.6 - 16.9), 20.4 weeks (18.6 - 22.5) and 30.2 weeks (28.5 - 32.5). The second and third visits were considered as second and third trimester measurements. These fetal ultrasounds were used both to establish gestational age (first ultrasound) and to assess fetal growth characteristics [22]. Fetal growth measurements used for the present study comprised head circumference (HC), abdominal circumference (AC) and femur length (FL) in the second and third trimester measured to the nearest millimeter using standardized ultrasound procedures [23-25]. Estimated fetal weight was calculated using the formula by Hadlock using head circumference, abdominal circumference and femur length: EFW (cm) = 10^(circ;1.326 - 0.00326*AC*FL + 0.0107*HC + 0.0438*AC + 0.158*FL) [26]. Growth measurements in early pregnancy (gestational age < 18 weeks) were not included, since these fetal ultrasound examinations were performed primarily to establish gestational age. Gestational age-adjusted standard deviation scores were constructed for these fetal growth measurements.

#### Postnatal growth characteristics

Birth weight, date of birth and gender were obtained from community midwife and hospital registries. Well-trained staff in community health centers obtained postnatal growth characteristics using standardized procedures. Based on the routine health care program, visits for these growth characteristics were grouped into three age periods: 6 (range 5 - 8.99) months; 14 (range 12 - 18.38) months and 24 (range 23 - 28.93) months. Anthropometrics were measured without clothes. Weight was measured to the nearest gram using electronic scales. Length was measured to the nearest millimeter in the supine position using a neonatometer at the ages of 6 and 14 months, and height was measured in the upright position at the age of 24 months. Head circumference was postnatally measured at the age of 6 and 14 months.

### Data analysis

Differences in baseline characteristics between boys and girls were examined by independent samples t-tests (continuous variables) or Pearson's chi-square (categorical variables). Because of the low number of homozygous subjects for haplotype 5 (n = 8) and 6 (n = 1), these haplotypes were analyzed as carriers (1 or 2 copies) and non-carriers (0 copies).

We used an additive model to study the effects of the different haplotypes on pre- and postnatal growth The associations of the glucocorticoid receptor haplotypes with pre- and postnatal growth characteristics (weight, length and head circumference) were analyzed in three different time-intervals: from second trimester to birth, from birth to 24 months of age and from second trimester to 24 months of age. Since, no data were available for head circumference at 24 months of age, head circumference was analyzed until the age of 14 months. With these outcomes, the effects of glucocorticoid receptor polymorphisms on skeletal and non-skeletal growth and head circumference can be studied. We used femur length as measure of skeletal growth in fetal life (correlation femur length in third trimester of pregnancy and length at 1 month of age: r = 0.30, p-value < 0.001). To assess longitudinally measured growth patterns from fetal life to infancy, we performed repeated measures regression analysis. This regression technique takes the correlation of multiple measurements within one subject into account, assesses both the time-independent and time-dependent effect of the glucocorticoid receptor genotypes, and allows for incomplete outcome data [27,28]. Haplotype and its interaction with age were included in these models. To account for age variation between individuals within each time interval, these analyses were conducted using age-adjusted standard deviation scores as outcomes. The models can be written as:

Similar models were used for length and head circumference growth. The term including 'β_0_' reflects the intercept and the term including 'β_1_' reflects the growth per week for the reference group. The terms including 'β_2_' and 'β_3_' reflect the age independent and dependent growth differences between the different categories of the glucocorticoid receptor genotype, respectively[28]. All betas, including the intercept and age interactions, were included as random effects in the models. To study the dominant effects of the glucocorticoid receptor gene polymorphisms, we merged the group heterozygous and homozygous variant subjects and performed the same analyses. In addition, we performed all these analyses stratified for birth weight.

Furthermore, we fitted multiple logistic regression models to analyze the associations of the different glucocorticoid receptor haplotypes with prenatal growth retardation (growth deceleration) and postnatal growth acceleration. We defined growth deceleration as a decrease in weight SDS from second trimester of pregnancy until birth of >-0.67 standard deviation and growth acceleration as an increase in weight SDS from birth to 24 months of age of more than 0.67 standard deviation [29]. Each anthropometric outcome was analyzed using gender and age adjusted standard deviation scores (SDS). These were based on reference growth curves from the whole study population. Age at visit and gender were included in these models as confounding variables, since they changed the effects estimates of interest on pre- and postnatal growth substantially (>10%).

With a sample size of 2,400 subjects and assuming a statistical power level (1 - β) of 0.80 and a level of significance (α) of 0.05, we were able to detect differences in growth characteristics of about 0.42 SD and 0.19 SD for exposure rates of 2% and 10%, respectively. These differences correspond with 246 and 112 grams in birth weight. Similarly we were able detect Odds ratios (OR) of 2.51 and 1.57 for exposure rates of 2% and 10%, respectively. All effect estimates are presented with their 95% confidence interval (95% CI). Statistical analyses were performed using the Statistical Package of Social Sciences version 15.0 for Windows (SPSS Inc, Chicago, IL, USA) and the Statistical Analysis System (SAS) for Windows, version 9.1.3.

## Results

The distribution of the different glucocorticoid receptor haplotypes within our study population is presented in Table 1. Haplotype 1, 2, 3 and 4 were most frequent with allele frequencies of 42.4%, 22.7%, 14.3% and 13.4%, respectively. Haplotypes 5 and 6 had allele frequencies of 4.1% and 3.1%, respectively. Comparison of means of baseline characteristics between carriers of 0, 1 or 2 copies of haplotype 1 to 6 revealed no significant differences for the covariates age at visit and gender. Table 2 presents the baseline characteristics of infants who participated in the postnatal visits. There was a slight male preponderance (51%) in our study population. The overall median ages (95% range) in infants at their visits were 6.2 months (5.2 - 7.9), 14.3 months (13.5 - 16.1) and 24.8 months (23.4 - 28.1).

**Table 1:** Distribution of the different haplotype alleles of the glucocorticoid receptor gene within our study population (n = 2,414)

Glucocorticoid receptor haplotype (copies)	N (%)
Haplotype 1	
0	531 (22.0)
1	1,379 (57.1)
2	504 (20.9)
Haplotype 2	
0	1,265 (52.4)
1	1,017 (42.1)
2	132 (5.5)
Haplotype 3	
0	1,668 (69.1)
1	699 (29.0)
2	47 (1.9)
Haplotype 4	
0	1,688 (70.0)
1	677 (28.0)
2	49 (2.0)
Haplotype 5	
0	2,196 (91.0)
1 or 2	218 (9.0)
Haplotype 6	
0	2,242 (92.9)
1 or 2	172 (7.1)

Values are number of persons (%).

**Table 2:** Fetal and infant characteristics (n = 2,414)

	Boys (n = 1,233)	Girls (n = 1,181)	P-value
**Second trimester**			
Gestational age (weeks)	20.6 (18.7 - 23.3)	20.4 (18.5 - 23.1)	<0.01
Estimated fetal weight (grams)	386 (91)	374 (86)	<0.01
Femur length (mm)	33.3 (3.2)	33.2 (3.3)	0.55
Head circumference (mm)	181.0 (13.1)	177.2 (13.0)	<0.01
**Third trimester**			
Gestational age (weeks)	30.4 (28.6 - 33.0)	30.3 (28.3 - 32.8)	<0.01
Estimated fetal weight (grams)	1646 (258)	1620 (268)	<0.01
Femur length (mm)	57.4 (2.9)	57.4 (2.9)	0.64
Head circumference (mm)	288.8 (11.8)	283.7 (11.2)	<0.01
**Birth**			
Gestational age	40.1 (35.0 - 42.4)	40.1 (35.2 - 42.1)	0.42
Weight (grams)	3518 (596)	3400 (574)	<0.01
Length (cm)*	55.1 (2.4)	54.0 (2.1)	<0.01
Head circumference)*	38.1 (1.3)	37.3 (1.2)	<0.01
Weight < 2500 grams (%)	29 (2.4)	26 (2.2)	0.80
Preterm birth (<36 weeks) (%)	41 (3.3)	37 (3.1)	0.79
**Age 6 months**			
Age at visit (months)	6.2 (5.2 - 7.9)	6.2 (5.2 - 7.9)	0.28
Weight (grams)	8,078 (845)	7,540 (812)	<0.01
Length (cm)	68.5 (2.4)	66.8 (2.3)	<0.01
Head circumference (cm)	44.2 (1.3)	43.1 (1.7)	<0.01
**Age 14 months**			
Age at visit (months)	14.3 (13.6 - 16.2)	14.3 (13.4 - 16.0)	0.05
Weight (grams)	10,824 (1,070)	10,191 (1,018)	<0.01
Length (cm)	78.9 (2.6)	77.5 (2.6)	<0.01
Head circumference (cm)	47.7 (1.3)	46.6 (1.1)	<0.01
**Age 24 months**			
Age at visit (months)	24.8 (23.4 - 28.1)	24.8 (23.4 - 28.1)	0.48
Weight (grams)	13,140 (1,376)	12,651 (1,412)	<0.01
Length (cm)	88.9 (3.4)	87.7 (3.4)	<0.01

Values are means (SDS) or medians (95% range).

Differences between boys and girls were compared using independent samples t-tests.

*Measured at 1 months of age

Table 3 shows the associations of glucocorticoid receptor haplotypes with pre- and postnatal weight until the age of 24 months. No consistent associations were found between the different haplotypes and repeatedly measured weight in the three different time-intervals. Also, no consistent associations were found with length and head circumference (Table 4 and 5). In the dominant models, the associations between the glucocorticoid receptor gene polymorphisms were also not significant. A stratified analysis among low birth weight children, focused on the associations of the haplotypes with fetal and early postnatal growth characteristics did not show any significant results (data not shown).

**Table 3:** Associations of glucocorticoid receptor haplotype with repeatedly measured weight from mid-pregnancy until 24 months of age

	Weight SDS change **2**^**nd **^**trimester - birth** (95% CI)	Weight SDS change Birth - 24 months (95% CI)	Weight SDS change 2^**nd **^trimester - 24 months (95% CI)
**Glucocorticoid receptor haplotype (copies)**			

Haplotype 1			
0	0.03 (-0.06, 0.12)	-0.02 (-0.12, 0.08)	0.05 (-0.05, 0.15)
1	0.02 (-0.05, 0.09)	-0.03 (-0.11, 0.05)	0.03 (-0.04, 0.11)
2	Reference	Reference	Reference
Haplotype 2			
0	Reference	Reference	Reference
1	-0.06 (-0.12, 0.01)	-0.02 (-0.09, 0.06)	-0.10 (-0.17, -0.03)*
2	-0.01 (-0.15, 0.13)	-0.01 (-0.17, 0.16)	-0.02 (-0.18, 0.15)
Haplotype 3			
0	Reference	Reference	Reference
1	0.06 (-0.01, 0.14)	0.06 (-0.02, 0.14)	0.09 (0.01, 0.17)*
2	0.02 (-0.21, 0.25)	0.02 (-0.26, 0.29)	0.14(-0.14, 0.42)
Haplotype 4			
0	Reference	Reference	Reference
1	-0.02 (-0.09, 0.05)	0.02 (-0.07, 0.10)	0.12 (-0.15, 0.40)
2	-0.21 (-0.43, 0.02)	0.07 (-0.21, 0.36)	0.16 (-0.10, 0.44)
Haplotype 5			
0	Reference	Reference	Reference
1 or 2	-0.01 (-0.18, 0.16)	-0.36 (-1.02, 0.29)	-0.16 (-0.75, 0.43)
Haplotype 6			
0	Reference	Reference	Reference
1 or 2	-0.20 (-0.41, 0.01)	0.97 (-0.99, 2.93)	0.54 (0.13, 0.96)*

Values are regression coefficients (95% confidence interval) and reflect the difference in standard deviation score of weight from 2^nd ^trimester until 24 months of age for the different glucocorticoid haplotypes. SDS, gestational age adjusted standard deviation score; CI, confidence interval. Models are adjusted for age at visit and gender.

**Table 4:** Associations of glucocorticoid receptor haplotype with repeatedly measured length from mid-pregnancy until 24 months of age

	Length SDS change **2**^**nd **^**trimester - birth** (95% CI)	Length SDS change Birth - 24 months (95% CI)	Length SDS change **2**^**nd **^**trimester - 24 months** (95% CI)
**Glucocorticoid receptor haplotype (copies)**			

Haplotype 1			
0	0.01 (-0.09, 0.10	-0.01 (-0.13, 0.12)	0.01 (-0.09, 0.10)
1	0.02 (-0.05, 0.09)	-0.01 (-0.10, 0.09)	0.01 (-0.07, 0.08)
2	Reference	Reference	Reference
Haplotype 2			
0	Reference	Reference	Reference
1	0.02 (-0.08, 0.12)	0.02 (-0.07, 0.11)	-0.07 (-0.14, 0.01)
2	0.08 (-0.08, 0.24)	0.03 (-0.19, 0.26)	0.10 (-0.06, 0.26)
Haplotype 3			
0	Reference	Reference	Reference
1	0.09 (0.02, 0.16)*	0.05 (-0.05, 0.15)	0.07 (-0.01, 0.14)
2	0.16 (-0.08, 0.40)	0.13 (-0.24, 0.50)	0.12 (-0.15, 0.38)
Haplotype 4			
0	Reference	Reference	Reference
1	-0.03 (-0.10, 0.05)	-0.07 (-0.17, 0.04)	-0.02 (-0.10, 0.06)
2	-0.23 (-0.47, 0.01)	-0.02 (-0.40, 0.34)	-0.15 (-0.42, 0.11)
Haplotype 5			
0	Reference	Reference	Reference
1 or 2	0.14 (0.02, 0.25)*	0.02 (-0.14, 0.19)	0.18 (0.05, 0.30)†
Haplotype 6			
0	Reference	Reference	Reference
1 or 2	-0.10 (-0.23, 0.03)	-0.05 (-0.23, 0.14)	-0.11 (-0.25, 0.03)

Values are regression coefficients (95% confidence interval) and reflect the difference in standard deviation score of length from 2^nd ^trimester until 24 months of age for the different glucocorticoid haplotypes. SDS, gestational age adjusted standard deviation score; CI, confidence interval. Models are adjusted for age at visit and gender.

**Table 5:** Associations of glucocorticoid receptor haplotype with repeatedly measured head circumference from mid-pregnancy until 14 months of age

	Head circumference SDS change **2**^**nd **^**trimester - birth** (95% CI)	Head circumference SDS change Birth - 14 months (95% CI)	Head circumference SDS change **2**^**nd **^**trimester - 14 months** (95% CI)
**Glucocorticoid receptor haplotype (copies)**			

Haplotype 1			
0	-0.10 (-0.20, 0.01)	-0.16 (-0.33, 0.01)	-0.09 (-0.18, 0.01)
1	-0.05 (-0.13, 0.03)	-0.11 (-0.24, 0.02)	-0.06 (-0.13, 0.01)
2	Reference	Reference	Reference
Haplotype 2			
0	Reference	Reference	Reference
1	-0.07 (-0.16, 0.01)	0.01 (-0.11, 0.12)	-0.05 (-0.12, 0.02)
2	-0.12 (-0.30, 0.06)	0.08 (-0.18, 0.35)	-0.02 (-0.17, 0.13)
Haplotype 3			
0	Reference	Reference	Reference
1	0.11 (0.03, 0.20)*	0.12 (-0.01, 0.25)	0.10 (0.02, 0.17)†
2	0.11 (-0.17, 0.40)	-0.12 (-0.53, 0.29)	0.08 (-0.16, 0.33)
Haplotype 4			
0	Reference	Reference	Reference
1	0.01 (-0.07, 0.10)	0.08 (-0.05, 0.21)	0.12 (-0.15, 0.40)
2	0.18 (-0.12, 0.47)	0.19 (-0.22, 0.60)	0.17 (-0.10, 0.44)
Haplotype 5			
0	Reference	Reference	Reference
1 or 2	-0.10 (-0.33, 0.13)	-0.17 (-0.38, 0.03)	0.02 (-0.10, 0.14)
Haplotype 6			
0	Reference	Reference	Reference
1 or 2	0.07 (-0.09, 0.22)	0.07 (-0.15, 0.29)	0.06 (-0.07, 0.19)

Values are regression coefficients (95% confidence interval) and reflect the difference in standard deviation score of head circumference from 2^nd ^trimester until 14 months of age for the different glucocorticoid haplotypes. SDS, gestational age adjusted standard deviation score; CI, confidence interval. Models are adjusted for age at visit and gender.

Associations of the different haplotypes with the risks of prenatal growth retardation (growth deceleration) and postnatal growth acceleration are presented in Table 6. No significant differences were found in risks of prenatal growth deceleration and postnatal growth acceleration for the different haplotypes. However, children who showed prenatally an increased risk of growth retardation compared to the reference, tend to have an increased risk of growth acceleration in postnatal life as well.

**Table 6:** Associations of glucocorticoid receptor haplotype with the risks of prenatal growth deceleration and postnatal growth acceleration until 24 months of age

	Prenatal growth deceleration (95% CI)	Postnatal growth acceleration (95% CI)
**Glucocorticoid receptor haplotype (copies)**	**Growth deceleration** **(>-0.67 SDS)**	**Growth acceleration** **(>0.67 SDS)**

Haplotype 1		
0	1.09 (0.84, 1.42)	1.07 (0.78, 1.48)
1	1.16 (0.95, 1.41)	1.11 (0.87, 1.42)
2	Reference	Reference
Haplotype 2		
0	Reference	Reference
1	0.88 (0.73, 1.07)	0.99 (0.79, 1.26)
2	1.03 (0.68, 1.55)	1.20 (0.73, 1.97)
Haplotype 3		
0	Reference	Reference
1	1.17 (0.95, 1.43)	1.05 (0.82, 1.35)
2	0.90 (0.45, 1.84)	0.42 (0.13, 1.39)
Haplotype 4		
0	Reference	Reference
1	0.88 (0.71, 1.09)	0.97 (0.74, 1.25)
2	0.93 (0.47, 1.84)	0.80 (0.27, 2.39)
Haplotype 5		
0	Reference	Reference
1 or 2	0.96 (0.69, 1.34)	0.96 (0.65, 1.42)
Haplotype 6		
0	Reference	Reference
1 or 2	1.26 (0.86, 1.87)	1.28 (0.80, 2.07)

Values are odds ratios (95% confidence interval) and reflect the difference in risk of prenatal growth deceleration and postnatal growth acceleration until 24 months of age for the different glucocorticoid haplotypes compared to the reference group (-0.67 to 0.67 SDS change in weight SDS). SDS, gestational age adjusted standard deviation score; CI, confidence interval. Models are adjusted for age at visit and gender.

## Discussion

In our population-based prospective cohort study we showed that glucocorticoid receptor gene polymorphisms are not consistently associated with growth in fetal and early postnatal life. Furthermore, we demonstrated that these polymorphisms were not related to size at birth or growth acceleration during the first 2 years of life.

The major strengths of our study are its prospective design from early fetal life and the size of the population-based cohort. Our analyses were based on over 6,000 growth measurements. Furthermore, the relative effect of variants of the glucocorticoid receptor gene on growth measurements might be larger in childhood, when the effect of various environmental factors, such as life style habits, is limited. Although, there are also studies that indicate that small differences between individuals may increase with advancing age [8,17]. In subjects who were born very preterm and followed up until the age of 19 years, the 23 K variant in the GR gene was associated with lower fasting insulin levels and a lower HOMA-IR as well as with a taller stature departing from the age of 1 year. Furthermore, these children showed complete catch-up growth between the ages of 3 months and 1 year. Apparently, the nature of the population studied also plays a role. A possible limitation is that the current study was performed in a relatively healthy cohort, resulting in a relatively small number of low birth weight children (n = 55). A stratified analysis among these children, focused on the associations of the haplotypes with fetal and early postnatal growth characteristics did not show any significant resuls. Larger studies among low birth weight children might be able to identify specific effects of haplotypes on early growth characteristics. DNA for genotyping was available in 59% of all subjects and was isolated from cord-blood. Missing cord-blood was mainly caused by logistical restraints at delivery. Of all genotyped eligible subjects at baseline, 22% did not participate in follow-up measurements. Our study was designed to assess pre- and postnatal growth in a relatively healthy group of children. As a consequence, the group of children born with small size for gestational age (n = 55) was too small for specific analyses focused on this group. Thus generalizability is limited with respect to children born preterm or with low birth weight.

Glucocorticoid receptor gene polymorphisms have been identified that contribute to the variability in glucocorticoid sensitivity. This sensitivity to glucocorticoids is known to show a large interindividual variation [1]. Persons vary considerably in their response to both endogenous and exogenous glucocorticoids. So it is likely that these polymorphisms are to some extent responsible for the variability in the sensitivity to glucocorticoids. Glucocorticoids are important regulators of the immune system, inflammatory processes and many other processes involved in fat and glucose metabolism. Previous studies examined the potential role of glucocorticoids in the development of adult disease. Studies in rats showed that activity of placental 11β-hydroxysteroid dehydrogenase type 2, which converts physiological glucocorticoids to inactive products, correlates positively with birth weight and negatively with placental weight[30]. Similar findings were found in small preterm infants [31]. Thus, fetuses with the greatest exposure to growth-retarding maternal glucocorticoids have low birth weight and high placental weight. In human studies, it is demonstrated that these fetuses might be at a higher risk of subsequent hypertension [32]. In addition, administration of low-dose dexamethasone to pregnant rats not only reduces birth weight but also leads to high blood pressure in young adult offspring [30]. Increased exposure to cortisol in adults leads again to low birth weight and postnatal growth acceleration, which are well-known risk factors for cardiovascular disease, type 2 diabetes and obesity [2,3]. Therefore, these polymorphisms in the glucocorticoid receptor gene could, by increasing glucocorticoid sensitivity in the fetus for maternal glucocorticoids, lead to intrauterine growth retardation and metabolic and cardiovascular diseases in adulthood. Genetically established differences between individuals in glucocortcoid sensitivity may also be associated with these diseases.

The effect of glucocorticoids is mediated by the glucocorticoid receptor. Rautanen et al reported a common glucocorticoid receptor haplotype to be associated with short length and low weight at birth and higher indices of HPAA function later in life [20]. In humans, the possible importance of glucocorticoid sensitivity on fetal growth and HPA programming has not been previously investigated. However, previous studies have examined the associations of different polymorphisms in the glucocorticoid receptor gene and sensitivity to glucocorticoids. The results of these studies are conflicting. A few studies report positive associations between the N363S and *Bcl*I polymorphisms and hypersensitivity to glucocorticoids, as was tested using a very low dose dexamethasone suppresion test (0.25 mg) [9-11], while other studies found no alterations of glucocorticoid sensitivity as tested with a low dose (0.5 mg) dexamethasone suppresion test [14]. The 23K variant of the R23K polymorphism was associated with relative resistance to glucocorticoids [7,8]. No associations were found yet with the *TthIII*I polymorphism [4,15]. These studies suggest that genetically established differences in glucocortcoid sensitivity are important for various growth, development and health related outcomes. In addition, it is known that environmental, dietary, and socioeconomic factors also play an important role in determinants of body composition and metabolic factors. Reported associations of genetic variants with growth outcomes depend on many additional factors, including differences in characteristics between populations, prevalence of the genotypes, and interactions. The distribution of the different glucocorticoid receptor haplotypes within our study population was similar as in the the general population [6,20]. Genotype and allele frequencies were in Hardy Weinburg equilibrium (p > 0.01). Furthermore, the distribution of genotype frequencies did not significantly differ between children with and without postnatal growth data avialable. Therefore we do not think that this would have introduced major bias. Gene-environment and gene-gene interactions might be important for glucocorticoid receptor genotypes. Such studies require much more power and need to be performed.

## Conclusions

We hypothezised that genetic variants leading to increased glucocorticoid sensitivity are associated with fetal growth retardation and postnatal growth acceleration. This hypothesis is based on previous observations showing associations of cortisol levels and low birth weight. Low birth weight and postnatal growth acceleration are again associated with obesity and other metabolic diseases. However, we did not find any consistent effect on pre- and postnatal weight, length and head circumference between the different glucocorticoid receptor haplotypes in our population-based study. Neither did we find associations with prenatal growth deceleration or postnatal growth acceleration.

## Competing interests

The authors declare that they have no competing interests.

## Authors' contributions

MG: planned the analysis, interpreted data, and draft the manuscript. ES: participated in the interpretation of the data and revised the manuscript for important intellectual content. JK: assisted in study design and interpretation of data, and critically revised the manuscript. HM, HR and HT: critically revised the manuscript. AH: conceived the overall study, secured funding, and critically revised the manuscript. VJ: participated in the design and coordination of the study, participated in the interpretation of data, and helped to draft the manuscript. All authors read and approved the final manuscript.

## Funding sources

The first phase of the Generation R Study is made possible by financial support from the Erasmus Medical Center, Rotterdam, the Erasmus University Rotterdam and the Netherlands Organization for Health Research and Development (ZonMw).

## Pre-publication history

The pre-publication history for this paper can be accessed here:

http://www.biomedcentral.com/1471-2350/11/39/prepub
